# Nickel-aluminium pincer complexes undergo cooperative bond activation

**DOI:** 10.1038/s42004-021-00519-w

**Published:** 2021-05-27

**Authors:** Andrew J. Bissette

**Affiliations:** Communications Chemistry, https://nature.com/commschem

## Abstract

Lewis acid additives such as aluminium can enable fascinating new reactivity in transition metal catalysts, but few catalytic intermediates have been characterised. Now, a nickel-aluminium pincer complex offers new mechanistic insight into transmetalation, and new potential for reactivity.

Heterobimetallic complexes of nickel and aluminium offer great potential for catalytic bond activation, but mechanistic understanding is limited by a lack of characterised intermediates. Now, Brendan Graziano, Matthew Vollmer, and Connie Lu from the University of Minnesota report an isolated nickel–aluminium pincer complex which not only offers new mechanistic insight, but points towards new modes of reactivity (10.1002/anie.202104050)^1^.

Bimetallic complexes can incorporate inverse dative bonds, in which a Lewis acidic ligand such as aluminium accepts electron density from a transition metal centre, in contrast to more conventional metal-ligand interactions. These Z-type interactions open up new catalytic reactions. But, with only a handful of transition metal–aluminium complexes characterised, particularly involving first-row elements, the mechanistic basis for such reactivity remains poorly understood. Building on their longstanding interest in bimetallic complexes, the Lu group previously^2^ reported examples of first-row transition metal–aluminium complexes stabilised by heptadentate ligands. While these ligands enabled characterisation of these species, they also constrained their potential. “The group 13 ion (in this case Al) is always buried and impacts the reactivity of the transition metal only indirectly by tuning the electronic properties of the transition metal,” explains Lu. “In this work, we use a simpler ligand platform so that substrates can access not only the Ni center but also the Al center directly. This enables both metals to work together directly in the cooperative activation of substrates while tapping into the unique properties of the Ni–Al bond.”

The simpler ligands used here are pincer-type ligands, which have enjoyed widespread application in catalysis, for example in small molecule activation. Obtaining stable complexes in which both metals are substrate-accessible, however, was a challenge. “We wanted the bimetallic complex to be open for coordination with the Lewis acidic site, but not so exposed that everything falls apart. There is always a balancing act between the most reactive design and the more well-behaved—our design strikes a good balance between both,” says Graziano. Treatment of a suitably balanced aluminium metalloligand with Ni(COD)_2_ yielded a dimeric bimetallic pincer complex, bridged by cyclooctadiene. As anticipated, studies of its reactivity showed that the open coordination environment of the aluminium cation allowed both metal centres to participate cooperatively in a range of stoichiometric reactions including with common sigma donors, C–X functionalisation, H_2_ cleavage, and oxidative addition of the ortho C–H bond in pyridine *N*-oxide.

Intriguingly, treatment of the dimeric pincer complex with THF yielded a product in which the aluminium-bound mesityl group had been transferred to the nickel centre (Fig. 1). The product has a very short nickel–aluminium bond, evidence for the formation of an aluminyl complex. Further exploration of this reaction by NMR spectroscopy supported a mechanism in which coordination of THF, or other O-donor ligands, to the aluminium centre causes cleavage of the metal-metal bond. “This aryl transfer somewhat mimics the transmetalation step found in cross-coupling chemistry,” says Graziano. “Transmetalation is often one of the most difficult and understudied steps in these reactions, and our system can be used as a model to shed light in this mechanism.”

**Fig. 1:**
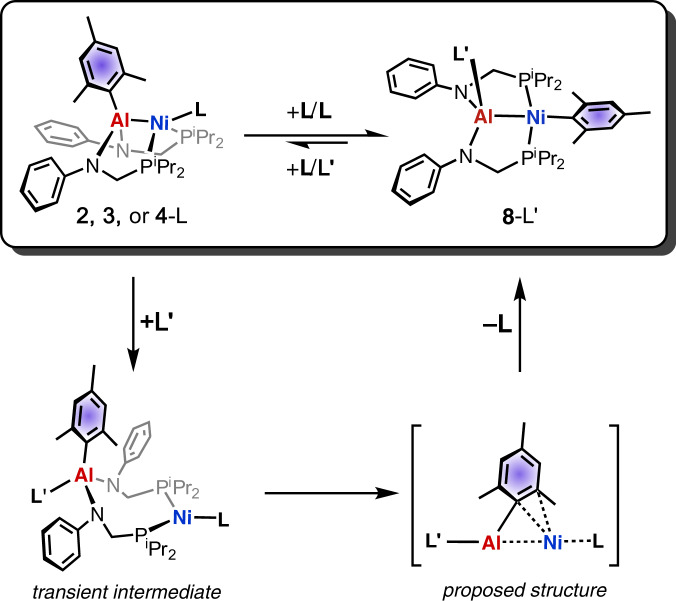
Aryl group transfer in a nickel–aluminium pincer complex. Top: Coordination of O-donor ligands, such as THF (denoted L), induces Al–Ni bond cleavage and aryl group (shaded blue) migration. The process is reminiscent of key transmetalation steps involved in cross-coupling reactions^1^. Copyright Wiley-VCH GmbH. Reproduced with permission.

The preliminary reactivity studies point to catalytic applications of these complexes. Homogeneous nickel hydrogenation catalysts are uncommon, for example. Lu is optimistic about their potential, saying, “This goal goes beyond replacing precious metals with a more sustainable alternative, but looking for opportunities to open new reactivity modes.”
